# Supplementary material for: Are carbon emissions trading and green financial instruments synergistic? -Comprehensive quantitative research based on content analysis
Source: PLoS One. 2024 Mar 7;19(3):e0298601. doi: 10.1371/journal.pone.0298601 (PMC10919601; doi:10.1371/journal.pone.0298601)
Supplement: S1 Data — (ZIP) [file pone.0298601.s001.zip › Data/地级市Z_F工作报告.zip!/─┌├╔╣┼/│α╖σ╩╨/└┤╫╘╣1⁄2╓┌║┼í╛┬φ┐╦╩2╛▌═°í┐_2011.docx]

各位代表：

　　现在，我代表市人民政府，向大会报告工作，请连同《赤峰市国民经济和社会发展第十二个五年规划纲要（草案）》一并审议，并请市政协各位委员和列席会议的同志提出意见。

　　一、十一五时期和2010年工作回顾

　　十一五时期是我市发展历程中极不平凡的五年。在市委坚强领导下，全市上下深入贯彻落实科学发展观，始终坚持发展第一要务，牢牢把握各种发展机遇，积极应对困难和挑战，求真务实，开拓进取，顺利完成十一五发展规划确定的目标任务，改革开放和现代化建设取得显著成绩。

　　（一）综合经济实力大幅提升。地区生产总值迈上千亿元台阶，年均增长17.7%；地方财政总收入突破百亿元大关，年均增长30.5%；固定资产投资累计完成2758亿元，是十五时期的4.6倍；城乡居民收入年均分别增长13.2%和12.2%。三次产业结构由2005年的26.5：36.8：36.7演进为16.4：51.1：32.5，实现了由农牧业主导向工业化引领的重大转变。农牧业综合生产能力稳步提升，已具备年产70亿斤粮食、45万吨肉类生产能力；新增设施农业50万亩，总面积达到66万亩；牧业年度家畜存栏稳定在1700万头只左右，连续五年保持全区第一。工业经济快速发展，年均增速达到26%，有色、能源、食品产业继续壮大，化工产业实现突破，机械制造业发展势头强劲；有色金属日采选、年冶炼能力分别由1.7万吨和17万吨提高到10.1万吨和64万吨，电力装机由166万千瓦增加到613万千瓦，大唐克旗煤制天然气、国电元宝山煤制尿素等一批重大化工项目落地实施；新增国家级高新技术企业7家，顺利通过国家知识产权试点城市考核验收，成为全国科技进步先进市；翁旗玉龙工业园区被认定为自治区首家承接产业转移示范园区，我市被命名为中国有色金属之乡。服务业发展提档升级，五年新增市场建筑面积600万平方米，5000平方米以上大型商业网点达到48家；农村牧区商贸网络日趋完善，消费环境不断改善；现代物流业快速崛起，红山、松山等物流园区蓬勃发展；旅游人数和旅游收入连年增长，2010年接待游客580万人次，实现旅游收入74亿元，克旗热水被命名为中国温泉之乡；金融机构存贷款余额较十五末分别增加560亿元和276亿元，金融生态环境持续改善。

　　（二）基础设施建设成效显著。区域性中心城市建设取得重要进展，中心城区建成区面积达到81平方公里、人口达到89万。新城区五年投入资金200.3亿元，实施重点建设工程200多项，城市路网、园林景观、商贸服务等基础设施和配套产业进一步完善。桥北组团和松山北城区开发建设全面启动，桥北组团核心区路网基本形成，松山北城区征地全部完成。老城区累计投入资金44.6亿元，改造扩建街路39条，治理小街小巷60条，新老城区实现协调发展。旗县政府所在地、重点小城镇建设快速推进，城镇综合承载能力和配套服务能力不断增强。全市城镇化率达到43.2%，年均提高2个百分点。生态保护与建设力度加大，五年投入资金48.4亿元，完成营造林面积993万亩，森林覆盖率提高3.2个百分点，达到34.01%，国家森林城市创建工作全面启动。交通、水利、电力等基础设施建设得到加强。完成公路建设投资137亿元，新建改建各级公路13732公里，其中高速公路311公里，实现了乡乡通油路、村村通公路。完成铁路建设投资59.4亿元，建成赤大白铁路，开工建设了赤锦、巴新、林白铁路，铁路运营总里程达到1101公里。建成新客运汽车站，对赤峰火车站、玉龙机场实施了改造。完成水利建设投资23.8亿元，建成德日苏宝冷、三座店、大石门子等一批重点水利工程，完成48座病险水库除险加固，解决了80.4万人饮水安全问题。完成电力投资68.1亿元，500千伏华峰宁输变电工程投入运行，新建改造变电站77座，每个旗县区至少拥有1座220千伏变电站，供电保障能力明显提升。

　　（三）社会事业全面发展。两基成果不断巩固，高中教育保持全区领先水平，中等职业教育迅速发展，民族教育得到加强，高等教育质量稳步提高；校舍安全工程扎实推进，中小学办学条件不断改善。投资15.7亿元，新改扩建医疗卫生机构257所，城乡公共卫生服务体系和医疗服务体系进一步健全；建成新型农村合作医疗市级信息平台，全市范围内实现异地就诊网上即时结报。成功举办五届红山文化节，圆满完成奥运火炬传递任务；启动辽代大遗址群申报世界文化遗产工作，赤峰博物馆新馆、玉龙文化产业园投入运营，巴林右旗被评为中国格斯尔文化之乡；新建苏木乡镇综合文化站74个、村级草原书屋1010个，城乡群众文化生活日益丰富。赤峰体育中心一期工程投入使用，二期工程加紧推进，获得自治区第十三届运动会承办权。全市人口出生率控制在11‰以内，稳定保持低生育水平。审计、监察、统计、外事、侨务、人防、气象、档案、老龄、残疾人、红十字、民族宗教、妇女儿童、食品药品、社区建设等工作取得新成绩。

　　（四）改革开放不断深化。农村牧区综合改革深入推进，集体林权制度改革主体任务基本完成。政府机构改革顺利实施，调整撤并了部分工作部门和办事议事机构。事业单位改革迈出新步伐，赤峰广播电台和电视台合并，赤峰日报传媒集团成立，组建了赤峰农牧科学研究院。完成农村信用社产权制度改革，引进包商银行重组赤峰城市信用社。财政收付制度改革不断深化，公务卡制度在市本级全面推行，村级公益事业建设一事一议财政奖补制度覆盖所有旗县区。医药卫生体制改革启动实施。狠抓招商引资和对外开放，在北京、沈阳、大连等多个大中城市成功举办了投资合作洽谈及重点项目推介活动，五年累计引进项目1721个，实际到位资金1489亿元，利用外资1.9亿美元。与大唐国际、京能集团、铜陵有色等大企业的合作不断深化，国电集团、云南铜业、山东黄金等一批外埠大企业与我市企业实现重组，新疆金风、华润集团、湖南有色等行业领军企业入驻赤峰。2010年，我市跻身中国十佳投资创业城市。

　　（五）民生状况持续改善。以实施惠民实事为抓手，着力改善群众生活，五年用于民生支出达到306.5亿元，占全市财政总支出的40.3%。累计新增城镇就业13.3万人，城镇登记失业率控制在4.2%以内，实现了零就业家庭至少一人就业的目标。劳务输出保持在100万人次以上，敖汉旗、巴林左旗被评为全国劳动力转移就业工作示范县。新农合参合人口313万，城镇居民基本医疗保险参保人数96.3万，城乡居民基本养老保险参保人数51.5万,28万职工纳入失业保险。企业退休人员人均月养老金标准五年提高711元，解决了3.5万名国有关闭破产企业退休人员医疗保险问题。城乡低保、农村五保户生活补助标准逐年提高，34.5万城乡困难群众的基本生活得到保障。全面启动保障性安居工程建设，累计建设经济适用房90万平方米、廉租住房29.8万平方米，改造农村牧区危房92万平方米，实施棚户区改造149万平方米，解决了5.4万户低收入家庭住房困难问题；发放住房公积金贷款35.6亿元，为3.3万户家庭提供了住房贷款支持。深入推进社会救济和扶贫开发，扶持31万贫困人口实现稳定脱贫。

　　（六）精神文明和民主法制建设不断加强。群众性精神文明创建活动蓬勃开展，社会文明程度进一步提高。成功承办了全区第六次精神文明建设经验交流会，充分展示了赤峰团结奋进的新形象和日新月异的新风貌。主动向人大及其常委会报告工作，向政协常委会通报情况，自觉接受人大依法监督和政协民主监督，累计办理市人大代表建议、意见596件，市政协委员提案1060件。认真听取各民主党派、工商联和无党派人士意见，决策科学化、民主化程度不断提高。五五普法全面完成，依法治市工作取得新成效。强化重点行业和领域安全监管，全市安全生产形势总体稳定、持续好转。信访工作不断加强，社会矛盾排查化解深入开展，集中解决了一批群众反映强烈的突出问题。加强社会治安综合治理，完善社会应急管理体系，依法打击各类犯罪活动，平安赤峰建设进一步巩固。深入推进国防动员和双拥共建，军地军民关系更加和谐。

　　各位代表，刚刚过去的2010年是十一五最后一年，也是赤峰市各项工作取得新成绩、开创新局面、迈上新台阶的一年。全市地区生产总值达到1080亿元，增长15%；地方财政总收入完成100.5亿元，增长22.2%；全社会固定资产投资848.7亿元，增长29.6%；社会消费品零售总额336.7亿元，增长19.4%；城镇居民人均可支配收入14108元，增长11.4%，农牧民人均纯收入5010元，增长11.3%。经过一年的努力，我们胜利完成了市五届人大四次会议确定的主要任务，为十一五划上了圆满句号。

　　回顾十一五，在推进经济快速发展和社会不断进步过程中，我们积累了许多宝贵经验，概括起来是：必须始终立足欠发达的基本市情，牢牢抓住发展这个第一要务，不断理清发展思路，创新发展理念，强化发展举措，在改革发展中解决各种矛盾和困难；必须坚持从实际出发，进一步发挥比较优势，加快构建多极支撑、多元发展的产业体系，不断推动经济社会发展实现新跨越；必须时刻关注民生，坚持把发展经济与造福百姓结合起来，使发展成果最大限度地惠及人民群众;必须勇于改革探索，创新体制机制，扩大对内对外开放，不断增强发展动力和活力；必须紧紧依靠全市各族人民，凝心聚力，团结奋进，大力营造干事创业的环境氛围，形成推动发展的强大合力。

　　各位代表，过去一年和十一五取得的可喜成绩，是市委正确领导、人大和政协监督支持的结果，是全市各族干部群众同心同德、齐心协力、顽强拼搏的结果，也是社会各界建言献策和鼎力相助的结果。在此，我代表市人民政府，向全市各族人民，向在各条战线上辛勤工作、付出努力的同志们，向所有关心和支持赤峰发展的朋友们，致以崇高的敬意和衷心的感谢！

　　在肯定成绩的同时，我们也清醒地认识到，我市经济社会发展中还存在着不少困难和问题。主要是：经济总量小，人均水平低，发展不平衡；农牧业基础设施薄弱，防灾减灾能力不强，现代化水平不高；产业结构不合理，经济增长对资源的依赖偏重，非资源型产业和中小企业发展缓慢，民间资本活力不足；城市基础设施建设与城镇化快速发展的客观要求不相适应，城镇管理水平亟待提高；生态环境依然脆弱，节能降耗、环境和水资源保护任务艰巨；城乡居民收入水平较低，农牧民持续增收难度较大；公共服务投入不足，改善民生任务繁重，社会稳定、安全生产等工作还需进一步加强；政府自身建设与人民群众的期望仍有差距，形式主义、官僚主义及奢侈浪费现象不同程度存在。对这些问题，我们将高度重视，并在今后的工作中认真加以解决。

　　二、十二五时期经济社会发展主要任务

　　十二五时期，是我市加快科学发展、推进富民强市、全面建设小康社会的关键时期，也是大有作为的重要机遇期。根据十七届五中全会和市委五届十四次、十五次全委会议精神，十二五时期，我市经济社会发展的指导思想是：坚持以邓小平理论和三个代表重要思想为指导，深入贯彻落实科学发展观，紧紧围绕富民强市目标，以科学发展为主题，以加快转变经济发展方式为主线，以保障和改善民生为出发点和落脚点，以改革开放创新为动力，加快新型工业化、城镇化和农牧业现代化进程，统筹城乡区域发展，加强生态、水资源保护和基础设施建设，大力发展社会事业，努力增强自主发展能力，促进经济持续快速健康发展和社会和谐稳定，为全面建设小康社会奠定坚实基础。经济社会发展的主要预期目标是：地区生产总值年均增长16%左右，地方财政总收入年均增长19%左右，城镇居民人均可支配收入和农牧民人均纯收入年均增长15%左右，各项约束性指标达到自治区要求。

　　（一）全力做大经济总量，促进经济持续快速发展。立足于欠发达的基本市情和发展不足这一主要矛盾，始终坚持发展第一要务，着力推动经济总量扩张。下大力量谋划和实施一批重大项目，完善项目推进的政策措施和工作机制，为经济发展提供有力支撑。把进一步扩大投资作为拉动内需、做大总量的重要抓手，加大对重点领域、薄弱环节的投资力度，发挥投资对经济增长的拉动作用。围绕建设有色金属产业基地、绿色农畜产品生产加工基地、新型能源与煤化工基地，做大做强优势特色产业，加快培育战略新兴产业，改造提升传统产业，实施百亿元园区、百亿元企业培育工程，创建国家级和自治区级工业园区，推动产业优化升级。巩固提高农牧业基础地位，大力发展现代农牧业，抓好百亿斤粮食生产能力、百万亩设施农业、百万头肉牛育肥和千万亩节水灌溉工程，做大做强产业化龙头企业，提高农牧业生产技术装备、规模化生产和社会化服务水平。大力发展生产性服务业，积极发展生活性服务业，提升旅游业发展水平，打造新业态、拓展新领域、培育新热点，实现服务业发展提质提速，比重和竞争力提升。

　　(二)转变经济发展方式，增强可持续发展能力。坚持在发展中促转变，在转变中谋发展，依靠增量调整结构，努力提高发展的协调性、全面性、可持续性。推进矿产资源合理开发与节约利用，促进探、采、选、冶、加一体化发展，提高资源综合开发利用水平。实行最严格的水资源管理制度，控制地下水开采，提高中水回用率，统筹农牧业、工业、城镇生活和生态用水，建设节水型社会。实施差别化土地政策，加强土地用途管制，节约集约利用土地。强化节能措施，支持节能新技术、新工艺推广和应用，构建循环型城镇和社区。加强规划指导和财税金融等政策支持，发展循环经济，建设循环经济园区。坚持生态立市战略不动摇，继续实施重点生态工程，认真落实生态补偿政策，严格实施围封禁牧等保护措施，进一步改善生态环境质量。倡导绿色、低碳、环保、文明、节约的生产和生活方式，保障可持续发展。建立以企业为主体、市场为导向、产学研相结合的科技创新体系，促进经济增长向依靠科技进步和管理创新转变。

　　（三）加强基础设施建设，提升发展保障能力。按照改老城、扩新区、修环路、保安居的发展思路，加快百万人口中心城市建设，拉大城市框架，提高城镇化水平，增强辐射带动能力，打造绿色、人文、宜居区域性中心城市。加快旗县政府所在地建设，进一步完善功能，增强承载能力，引导产业向园区集中、人口向城镇集聚。因地制宜，突出特色，建设一批工贸结合型、交通枢纽型、文化旅游型重点小城镇。搞好社会主义新农村新牧区规划建设，推动城镇基础设施向农村牧区延伸，促进城市公共服务向农村牧区覆盖，提高农村牧区公共服务水平，努力实现以城带乡、城乡互动、协调发展。加快交通和能源通道建设，建成适应生产生活需要的现代化交通运输体系和电力、燃气等输送网络。加强水利基础设施建设，推进灌区续建配套与节水改造，有效解决我市资源性、工程性缺水问题。高度重视信息基础设施建设，在经济发展、社会管理和民生服务中广泛应用信息技术，不断提高数字赤峰建设水平。

　　（四）推进改革开放，增强经济社会发展动力和活力。加快发展非公有制经济，营造环境，拓宽领域，发挥其繁荣经济、活跃市场、拉动就业的作用。支持我市企业与央企、外企和大型民营企业联合重组，提高企业市场竞争力。转变政府职能，改进管理方式，提高行政效率和服务水平，推进法治政府和服务型政府建设。加快推进财税金融体制改革，提高财政资金的使用效益和金融对经济社会发展的支撑作用。深化资源性产品价格和资源配置方式改革，实施好农村牧区综合改革和配套改革，全力推动医药卫生体制改革，稳步推进教育、科技、文化、体育等事业单位分类改革，不断增强经济社会发展活力。加快与周边地区的互利合作，促进资源跨区域配置，推进重点产业和重大基础设施项目对接，共同打造产业带和经济区。主动接受京津冀、辽中南城市群辐射，加强与北京市全方位合作，扩大向沿海及经济发达地区开放。拓展合作方式和领域，加快承接产业转移园区建设，吸引更多的发达地区企业和项目入驻，在引资和引智上实现更大突破，使我市获得新的后发优势和更大发展空间。

　　（五）坚持富民与强市并重，努力建设和谐赤峰。实施更加积极的就业政策，加快发展劳动密集型产业、微小企业和服务业，培育就业增长点，推动以创业带动就业，保持城乡就业稳定增长。推动城乡居民收入普遍较快增加，努力实现居民收入增长和经济发展同步，劳动报酬增长与劳动生产率提高同步。推进覆盖城乡居民的社会保障体系建设，在扩大覆盖范围、提升保障水平、提高统筹层次等方面迈出更大步伐。推进保障性安居工程建设，改善低收入群体住房条件。组织开展多种形式的社会救助活动，解决困难群体的生活问题。推动各级各类教育协调发展、城乡教育均衡发展，促进教育公平，三年内实现高中免费教育。坚持公共医疗卫生的公益性质，加强公共卫生服务体系、医疗服务体系、医疗保障体系和药品供应体系建设，促进人人享有基本医疗卫生服务。大力发展文化事业，加快发展文化产业，推进文化创新，提升文化软实力。加快体育基础设施建设，推进群众体育、竞技体育和体育产业协调发展，认真筹备、高水平承办自治区第十三届运动会。推动社会管理创新，健全社会矛盾排查化解长效机制。加强社会应急管理，抓好安全生产和食品药品监管。推进平安赤峰建设，加强社会治安综合治理，依法严厉打击各种违法犯罪活动，营造和谐稳定的社会环境。

　　经过五年努力，全市综合经济实力显著增强，发展水平和质量明显提高；多元发展、多极支撑的现代产业体系基本形成，结构调整和经济发展方式转变取得重大进展；生态环境持续好转，水资源实现合理配置和高效利用，基础设施条件明显改善，城乡面貌发生较大变化；覆盖城乡的基本公共服务体系初步形成，社会建设和管理水平显著提高；人民群众生活质量进一步改善，健康幸福指数大幅提升，在全面建设小康社会的道路上迈出坚实步伐。

　　三、2011年主要工作安排

　　今年是十二五开局之年，也是中国共产党成立九十周年，做好今年的工作至关重要。经济社会发展主要预期目标是：地区生产总值增长17%；地方财政总收入增长20%；全社会固定资产投资增长21%；社会消费品零售总额增长18%；城镇居民人均可支配收入和农牧民人均纯收入分别增长15%；完成自治区下达的各项节能减排任务。

　　实现上述目标，要重点抓好以下工作：

　　（一）着力发展工业经济，推动产业结构优化升级

　　坚持工业优先发展、率先发展不动摇，坚定不移地实施工业强市战略，推动工业经济优化结构、提速增效，带动全市经济实现快速健康发展。

　　做大做强资源型产业。抓好有色、能源、食品、化工、建材等优势特色产业，做大产业规模，提升产业层次。支持国维、金域、红岭等矿山企业进一步扩大采选规模，加快山金10万吨铅冶炼、亚鼎10万吨铜材加工等项目建设，推动东北特钢2万吨钼制品加工、中色10万吨锌冶炼、富邦铜业10万吨铜冶炼项目投产达效，有色金属产业年销售收入达到700 亿元。开工建设中电投2×30万千瓦热电机组，推进大唐2×100万千瓦火电、京能二期2×30万千瓦热电前期工作；抓好罕山、乌套海、灯笼河等风电项目，新增风电装机60万千瓦。加快大唐煤制气、国电煤制尿素、双赢化工磷酸二铵等项目建设，推动绍根煤田重组取得实质性进展。推进冷山糖业2000吨干酵母、草原兴发1500万只肉鸡屠宰加工项目，鼓励宁城老窖、健元鹿业等食品加工企业加快新产品研发，打造绿色、有机农畜产品品牌。完成阿旗山水水泥日产4800吨熟料扩建，推动喀旗青山100万吨水泥开工建设，加快开发新型建筑材料，壮大建材产业。积极争取国家、自治区地勘资金，推进敖包吐、萨力巴、大坝东沟等风险探矿，加大重点矿区外围和深部找矿力度，增强后备资源保障能力。

　　大力培育非资源型产业。积极发展机械制造、绿色能源、生物制药、电子信息等产业，促进产业多元发展。抓好金成重工矿山设备制造、国电风机叶片制造及总装等项目，推动克旗京城风机总装、翁旗海滨矿山机械制造项目开工建设。加快推动宁城1500吨多晶硅项目落地实施，确保阳光科技1000吨光伏材料项目投产达效。抓好松山海嘉诺医药发酵基地、元宝山宁夏伊品氨基酸系列产品生产等项目建设，进一步做大生物制药产业规模。抓好五甲万京信息科技产业园建设，推动电子信息产业加快发展。

　　下大力气抓好工业园区建设。按照产业集群、项目入园的总体要求，整合资源，统筹规划，完善条件，最大限度地共享水电路等公共设施，推动企业入园发展、项目入园建设、产业集中连片。完善赤峰经济开发区管理体制机制，统筹抓好一区三园招商引资，推进远联三期100万吨钢、金剑10万吨电解铜、九联煤化120万吨捣固焦等项目建设，提高整体竞争力，建设自治区新型工业化产业示范基地，力争进入国家级开发区行列。按照产业配套、产业延伸、循环发展的原则，加快建设克旗煤化工、右旗煤电化、翁旗承接产业转移、喀旗铜加工、红山农畜产品加工、宁城机械制造等特色园区，构建铜深加工、风电、化工、食品、装备制造等产业集群，增强园区发展后劲和集聚发展能力。

　　加快发展中小企业。落实国家、自治区促进中小企业发展的各项政策，加大贷款贴息、税费减免、专项投入等政策支持力度。制定和完善促进中小企业发展的扶持措施，推动、引导中小企业向专、新、特、精方向发展，实现以小补大、以专配全，打造产业集群。继续增加小额贷款公司和地方性金融机构，提高中小企业授信等级，拓宽中小企业融资渠道。完善赤峰市中小企业信息网，建设中小企业服务大厅，为中小企业提供技术、政策、法律、信息和融资等服务。

　　（二）夯实农牧业基础，加快发展现代农牧业

　　坚持把兴农兴牧作为稳市之本、富民之基，毫不放松地抓好三农三牧工作，推动农村牧区经济持续发展、农牧民稳定增收。

　　增加对农牧业的投入。落实中央各项强农惠农政策，确保财政支出重点向农牧业和农村牧区倾斜，预算内固定资产投资重点投向农村牧区，土地出让收益重点用于农牧业土地开发、农田水利和农村牧区基础设施建设。鼓励和支持龙头企业、工商企业投资农牧业，引导社会资本向农牧业和农村牧区流动。加大涉农涉牧信贷投放力度，保持涉农涉牧贷款增量占比不低于上年，增速不低于同期各项贷款增速。加大各类支农支牧资金整合力度，集中力量建设重大农牧业工程项目。

　　加强农田水利基础设施建设。完成全市水资源规划工作，搞好水资源调查评价，掌握水资源变化及其开发利用情况，推进水资源科学合理和可持续利用。大力推广喷灌、滴灌和管灌等先进适用节水灌溉技术，完成优质牧草节水灌溉10万亩，建设节水灌溉经济林22万亩，发展玉米膜下滴灌100万亩，完成大中型灌区续建配套和节水改造年度投资1亿元。抓好小型病险水库、水闸除险加固，加快中小河流治理和山洪地质灾害防治工作。实施打虎石水库库区及移民安置区坡改梯综合试点项目，做好达里诺尔湖水生态及周边环境修复工程前期准备工作。大力发展民生水利，筹措资金2.3亿元，建设饮水安全工程540处，解决30万人、44万头只牲畜饮水安全问题。加强中心城区水源地建设和管理，提高用水保障能力。组织实施好第一次全国水利普查工作。

　　提高农牧业综合生产能力。推进百亿斤粮食生产能力建设，搞好农业综合开发和土地整治，加快建设旱涝保收高标准农田，抓好玉米、马铃薯等优势作物和杂粮杂豆等特色作物种植，促进粮食稳定增产。继续推进百万亩设施农业建设，创新建设管理机制，新增设施农业10万亩。抓好百万头肉牛育肥工程，加强种源基地和良繁体系建设，新建一批标准化养殖场、育肥小区和育肥专业村。完成昭乌达肉羊育种验收工作，打造地方肉羊品牌。

　　推进农牧业产业化进程。支持塞飞亚、草原兴发等企业扩产提效，推动东方万旗等企业实现重组，抓好博天糖业100万吨甜菜、燕京啤酒5万吨麦芽等产业化项目，继续引进一批新的产业化龙头企业，提升龙头企业规模和实力。支持上京、玉龙、文钟、平庄等农畜产品加工园区加快发展，推动农畜产品加工企业向园区集中。大力发展农牧民专业合作组织，加快建设肉牛、肉羊、生猪、家禽以及蔬菜、杂粮、药材、花卉等专业村和标准化生产基地，满足龙头企业原材料需求。加强动植物疫病防控，完善农畜产品质量监管体系，推进农牧业标准化生产，保证农畜产品质量安全。

　　加快新农村新牧区建设。加强农牧民最急需的生活基础设施建设，抓好村庄道路、改水改厕、垃圾处理、绿化美化等工程，进一步改善农村牧区生活环境和村容村貌，解决农牧民在饮水、行路、用电等方面的困难。加强宅基地规划和管理，节约村庄建设用地。加快发展农村牧区社会事业，开展劳动力技能培训，培养造就有文化、懂技术、会经营的新型农牧民。精心组织，专项推进，建设一批新农村、新牧区示范村。

　　（三）加快发展服务业，壮大服务业规模

　　坚持把服务业作为优化经济结构、加快转变经济发展方式的重要着力点，完善政策，强化措施，推动服务业扩体增量，提档升级。

　　发展壮大现代物流业。完成红山物流园区石油仓储库、冷链物流、青年物流信息创业大厦等重点项目建设，积极发展大宗商品电子交易、期货交易、服务外包等新兴业态，年交易额达到120亿元以上。推进松山物流园区雨润农副产品交易配送中心建设，完成投资15亿元。推动和美园区商贸城、餐饮娱乐城项目建成运营。发挥交通汇聚优势，大力推进大板物流园区建设。统筹抓好旗县物流园区和配送中心建设，打造煤炭、粮食、蔬菜等物流集散地。以公路、铁路沿线为重点，培育发展商贸物流产业带。

　　提升旅游业发展水平。加快阿斯哈图、美林谷、亲王府、玉龙沙湖等骨干景区景点建设，推动黄岗梁滑雪场等重点旅游项目落地开工，打造旅游品牌。统筹抓好克旗热水、敖汉华夏第一村温泉旅游度假区、红山旅游商贸城等餐饮、住宿、购物、娱乐配套设施建设，提升旅游接待能力和水平。引进有实力企业，开发文化旅游资源，研发特色旅游产品，提升旅游产业素质。加强旅游培训，强化旅游监管，提升旅游服务水平。抓好旅游宣传推介，开拓东北、环渤海、长三角等区域客源市场，打造绿色旅游通道，吸引更多游客到我市旅游。全年接待游客640万人次，实现旅游收入88亿元。

　　促进商贸服务业提档升级。抓好中心城市商圈建设，加快金钰大都会二期、维多利购物中心、印象红山、东方永业城市广场建设步伐，发展电子商务，提升商贸服务业档次，扩大辐射范围。推进旗县大型商场、高档宾馆和娱乐服务项目及城市综合体建设，增强服务功能，优化消费环境。发展家政服务、社区服务、家庭用品配送等便民服务业，健全社区服务网络。推进万村千乡市场工程和新网工程建设，新建和改造市县两级日用消费品、农资配送中心12个、村级连锁农家店320个，完成15处农畜产品市场升级改造。鼓励方便生产生活的连锁企业向城镇社区和农村牧区延伸，做好家电、汽车、摩托车下乡工作，拓展农村牧区消费市场。

　　加快发展金融业。引导金融机构加大对三农三牧、承接产业转移项目、中小企业和民生工程的信贷支持力度，全年信贷投放总额增长15%以上。推动金融企业增设旗县区分支机构，加快引进新的股份制商业银行，积极推动赤峰农村商业银行筹建工作，加快发展小额贷款公司。支持融资平台增资扩股，规范发展，增强担保实力，扩大担保规模，推进担保品种、抵押方式创新。探索建立新能源、有色金属、绿色农畜产品等产业私募基金，积极培育股权投资市场，推动汇风新能源、凌志食品等企业上市融资。支持保险业和证券业发展，引导有条件的企业进入国内外资本市场，探索发行中小企业集合债券、票据及短期融资券。加强诚信赤峰建设，维护金融生态安全。

　　（四）加强城乡基础设施建设，改善经济社会发展条件

　　加快推进城镇化步伐，加强生态保护与建设，统筹推进交通、水利、电力等各领域基础设施建设，为经济社会发展提供有力支撑。

　　加强中心城区规划建设和管理。完成《赤峰市城市总体规划》修编，加快中心城区专项规划编制，扩大控制性详细规划覆盖面，提高修建性详细规划编制水平，强化规划执行，加大执法监督力度。加快小新地组团征地拆迁和有序开发，完善八家组团路网、景观工程及学校、医院和商贸服务等配套设施。实施桥北组团行政区、商业区、文体中心和滨河景观带建设，完成65万平方米商住楼主体工程，确保37万平方米回迁安置房分期交付使用。加快松山北城区三横四纵主街路、行政小区、回迁安置小区、京通铁路公铁立交桥等工程建设，集聚人气商气。启动城区中环路建设，缓解中心城区交通压力。高度重视老城区改造和城市管理工作，把改造老城区和加强城市管理摆到与新区建设同等重要位置。加快老城区城中村、城边村改造及道路、桥梁、市政公用设施建设，完成100万平方米老旧小区改造。修改完善《赤峰市城市管理暂行办法》，推行数字化、精细化管理模式，开展城市环境综合整治，高水平管理城市。

　　提高城镇建设水平。加强旗县城关镇和重点小城镇规划编制，发挥规划在城镇建设和管理中的指导作用。抓好旗县政府所在地开发建设，完成一批街路、公园、绿地及棚户区改造等重点工程。加大对环境卫生、市容市貌等综合整治力度，打造高标准示范街路。实施旧小区改造，着力完善地下管网、绿化硬化和配套服务设施。推进34个重点小城镇建设，打造特色鲜明的精品小城镇。积极发展县域经济，强化城镇产业支撑，引导农村牧区人口加快向城镇转移。全市城镇化率提高1.5个百分点，达到44.7%。

　　强化生态建设与保护。推进京津风沙源治理、三北防护林建设、低产低效林改造、水源涵养林建设等重点生态工程，完成营造林面积302万亩。加快国家森林城市创建，投资4.6亿元，抓好35个重点建设项目，新增绿化面积16.5万亩。大力培育木材加工、林下经济、森林沙漠生态旅游、林木种苗花卉等生态产业，促进生态、经济、社会效益相统一。落实草原生态保护补助奖励政策，强化封育禁牧、舍饲圈养、生态移民等保护措施，严厉打击非法开荒、毁林毁草等行为，巩固生态建设成果。继续做好节能减排工作。

　　改善交通、电力基础条件。确保赤朝高速公路、林西至省际通道一级公路年内竣工通车，加快推进赤承高速公路、赤峰至凌源一级公路建设，开工建设克旗热水至林西一级公路。加快通村油路建设，实现红山区、元宝山区、喀喇沁旗村村通油路。加快赤锦、林白、巴新铁路建设，开工赤峰至京沈客专连接线、集通铁路复线和白浩铁路建设，推进赤大白新增二线扩能前期工作。完成玉龙机场1.9万平方米停机坪扩建，增加赤峰到北京等大中城市航班。投资19.7亿元，完成金厂沟梁、锦山、绍根、经西、林东、罕山220千伏及中心城区头道街、彩虹桥66千伏等输变电工程建设，提高供电保障能力。

　　（五）扎实推进各项改革，提高对内对外开放水平

　　坚持以改革开放增强经济社会发展的动力和活力，不断创新和完善与科学发展相适应的新体制新机制，努力构建多层次、宽领域、全方位的对内对外开放格局。

　　推进重点领域改革。深化农村牧区综合改革，加快土地和草牧场流转，发展多种形式的适度规模经营。推进集体林权制度改革，基本完成林权证发放。完善市本级公务卡使用管理，开展旗县区公务卡制度试点，完善村级公益事业建设一事一议财政奖补制度。推进事业单位分类改革和绩效工资制度改革，创新事业单位管理机制。推行非基本公共服务市场化改革，积极引进社会资本，增强社会服务供给能力。推进医药卫生体制改革，落实国家基本药物制度，完成239所卫生院、57个公立社区卫生服务机构综合改革。深化市政公用事业改革，扩大城市公用事业特许经营范围。

　　扩大对内对外开放。加快与相邻盟市及周边地区的互利合作，推进重点产业和重大基础设施项目对接，提高区域合作水平。面向京津冀辽鲁等区域，积极承接以纺织、服装、鞋帽为主的劳动密集型产业，有色金属、非金属、化工为主的精深加工产业，矿山机械、小五金、农机具、零配件加工为主的机械制造业和整体配套产业转移，提升发展层次。加强与北京市全方位合作，突出抓好产业对接，提升产业合作水平，增强自我发展能力。加强与进驻我市大企业、大集团战略投资合作，加快引进新的战略投资者。全年引进资金585亿元，增长22%。支持外贸企业优化出口结构，开拓外贸市场，完成进出口总额5.7亿美元，增长6%。

　　支持非公有制经济发展。落实资金、税收、用工等扶持政策，减轻企业负担，为非公有制企业发展创造宽松环境。发挥中小企业投资公司和担保公司的融资担保平台作用，解决非公有制企业融资难题。拓宽非公有制经济发展领域，引导非公有制企业围绕大企业、大集团生产加工初中级产品，开展包装、储运等服务。支持非公有制企业加快结构调整，推动技术进步和体制机制创新，增强可持续发展能力和核心竞争力。重视培育本土企业家，扶持本土企业加快发展，鼓励赤峰籍有识之士回乡创办企业。

　　（六）重视发展科教事业，增强人才和智力保障

　　坚定实施科教兴市战略，推进科技创新和各级各类教育协调发展，推动人口大市向人力资源和人才大市转变，为经济社会快速发展提供强有力的智力支持。

　　推动科技进步。筹建蒙药中药等技术创新战略联盟，新增国家级高新技术企业和自治区级企业技术研发中心5家。进一步完善规划，整合元宝山和润等农业科技资源，推进国家农业科技园区建设，发挥示范带动作用。围绕优势特色产业，抓好科技项目库建设，促进科技与经济紧密结合，为结构调整和产业升级提供科技支撑。做好知识产权试点市工作，争取进入国家知识产权示范城市创建市。办好内蒙古第九届农牧业科技成果博览会，扩大影响，提升档次。

　　加快发展教育事业。积极发展学前教育，加强公办园建设管理，鼓励和规范民办园发展，提高学前教育入园率和保育教育质量。巩固两基工作成果，加强义务教育阶段师资队伍建设，提高基础教育发展水平。加快启动宁城高级中学、八里罕中学、乌丹一中、林东一中易址新建和大板三中扩建项目，优化教育布局，推动普通高中特色化、均衡化发展。逐步推行高中免费教育，从今年起全部免除蒙古语授课普通高中和中等职业教育学生的学费和课本费，免除汉语授课普通高中困难家庭学生的学费和课本费。大力发展职业教育，完成赤峰一职专迁建工程，加强华夏职校、宁城职教中心、赤峰卫校、内蒙古交通职业技校等实训基地建设。重视和支持高等教育，办好赤峰工业职业技术学院，优化赤峰学院、内蒙古交通职业技术学院专业设置，进一步提高高等教育质量。落实优先发展民族教育各项政策措施，支持特殊教育发展，新建特校4所。抓好校安工程建设，完成193万平方米新建、改造、加固校舍任务，确保三年规划任务高标准、高质量完成。

　　强化人才保障。深入实施玉龙人才工程，统筹抓好党政人才、专业技术人才、企业经营管理人才、高技能人才、农村牧区实用人才、社会工作人才队伍建设。围绕重要领域、重点行业、重大项目和重点学科建设，大力培养和引进能够提高我市自主创新能力的领军人才、学术技术带头人及其后备力量,优化人才结构。创新人才管理和使用机制，完善人才资源市场化配置机制，促进人才合理流动。

　　（七）推动文化繁荣发展，提高文化软实力

　　深入挖掘和弘扬我市优秀的历史文化和民族文化，大力发展文化事业和文化产业，更好地满足人民群众日益增长的精神文化需求，提高文化对经济社会发展的推动力和影响力。

　　提高群众文明素质。加强社会公德、职业道德、家庭美德、个人品德建设，引导人们知荣辱、讲正气、尽义务。开展对网络、网吧、出版物市场和校园周边环境的集中整治，支持关心下一代工作，抓好未成年人思想道德实践活动，促进未成年人健康成长。启动全国文明城市创建工作，深入开展文明单位、文明村镇创建活动，推动优秀文化进学校、进机关、进农村牧区、进社区、进企业，进一步提高全社会文明程度。广泛开展赤峰精神大讨论和表述语征集活动，激发全市人民热爱家乡、共建美好家园的精神动力。全面启动六五普法，扎实开展依法治市工作，提高全社会知法、懂法、守法、用法水平。

　　大力发展文化事业。推进文化惠民工程，投资6600万元，新建综合文化站86个、草原书屋800个，进一步改善基层文化基础设施。完成锦山文化艺术中心和宁城、右旗、阿旗博物馆建设。抓好文艺精品创作，推出一批展示地方文化特色的新作品。加大文化遗产保护与利用，完成二道井子文化遗址保护规划，推进深度考古发掘及遗址公园项目建设。继续做好辽代大遗址群申报世界文化遗产工作，力争辽上京、辽陵及奉陵邑国家考古遗址公园申报立项获得国家批复。推动文化信息资源共享工程向农村牧区延伸，实施农村牧区电影2131工程，完成广播电视20户以下自然村村村通工程。加快广播电视网络建设，扩大数字广播电视覆盖面。

　　培育壮大文化产业。坚持挖掘弘扬历史文化、做大做强文化品牌、有序提升娱乐文化、培育发展创意文化，完成全市文化产业发展规划。落实国家、自治区和我市促进文化产业发展的优惠政策，加大对文化产业扶持力度，开工建设赤峰文化产业园和兴隆洼文化旅游产业园，完善巴林石文化创意产业园功能，搞好巴林石雕刻、展览和营销。放宽市场准入，积极培育和引进骨干文化企业和战略投资者，发展演艺娱乐、文化创意、文博会展、网络信息等特色文化产业，提升工艺美术、礼仪广告、出版印刷、文化旅游、体育休闲等传统文化产业水平。办好红山文化节、草原文化旅游节、巴林石节等节庆活动，打造节庆品牌。

　　（八）千方百计改善民生，扎扎实实为民办事

　　把保障和改善民生作为各项工作的出发点和落脚点，不断强化民生认识，落实民生政策，实施民生工程，让人民群众共享改革发展成果。

　　积极促进就业。加强技能培训和就业指导服务，完成3.2万人职业技能培训。突出抓好大中专毕业生、农村牧区剩余劳动力和困难群体、特殊群体就业工作，动态消除城镇零就业家庭。积极创建国家级创业型城市，鼓励支持自主创业和自谋职业，在信贷、税费、培训等方面加大扶持力度，促进以创业带动就业。筹措资金4000万元，开发公益岗位3000个，新增城镇就业1.8万人。

　　增加城乡居民收入。健全企业职工工资正常增长机制，提高社会保障水平，稳步增加城镇居民收入。筹措扶贫资金2.3亿元，扶持6万低收入人口稳定增收。挖掘农牧业内部增收潜力，落实惠农惠牧政策，鼓励农牧民向二三产业转移，不断增加农牧民生产性、工资性、转移性收入。高度重视牧民贫困问题，认真落实自治区对牧民生产生活的补贴政策，积极探索舍饲圈养、良种繁育、转移就业的扶持措施和禁牧、休牧、轮牧的补偿机制，促进牧民收入稳定增长。

　　提高全民健康水平。健全城乡医疗卫生服务体系，完成赤峰学院附属医院、松山区医院迁址新建和红山区医院病房楼建设工程，新建林西县医院、敖汉旗医院、元宝山医院、市精神病医院综合病房楼，改造村卫生室50个。建设市级医疗卫生应急救援指挥中心，完善全市急诊急救网络和视频应急指挥系统，提高急诊急救和突发性公共卫生事件应急处置能力。支持蒙医中医事业加快发展。大力开展爱国卫生运动和健康教育，做好国家卫生城市复审迎检工作，巩固国家卫生城市创建成果。推进全国人口和计划生育综合改革示范市建设，抓好生育关怀和出生缺陷预防，提高出生人口素质。加快市体育中心二期建设，做好全市第二届运动会筹备工作，广泛开展全民健身运动，提高城乡居民健康水平。

　　增强社会保障能力。做好企业基本养老保险自治区级统筹工作，继续提高企业退休人员基本养老金待遇水平。扩大国家新农保试点范围，确保45万农村牧区人口参加新农保。抓好基本医疗保险市级统筹、城乡一体和商业保险参与城乡医保试点工作，进一步提高城乡居民医疗保障水平。新增城镇基本医疗保险1万人，新农合住院报销封顶线提高到5万元。扩大失业、工伤和生育保险制度覆盖面，积极筹划工伤保险市级统筹。提高城乡居民最低生活保障标准，城镇低保月提高30元，农村牧区低保年提高180元。落实各项救助政策，保证五保供养和孤儿供养补助资金及时足额发放。做好困难家庭大中专学生资助和困难群体救助工作，扩大贫困残疾人救助面。发展老龄事业，筹建赤峰市老年护理院，向老年人提供日间照料、托管、康复等养老护理服务。

　　做好住房保障工作。严格落实国家房地产市场调控政策，改革住房用地供应方式，增加保障性住房和中小套型普通商品房供应，合理引导住房需求，把房价控制在合理水平。加强保障性安居工程建设，全年投资37亿元，建设公共租赁房、廉租住房等保障性住房2.2万套、130万平方米，改造农村牧区危旧房1万户、60万平方米。加强房屋建筑质量监管，严格执行住宅小区建设标准和配套标准，提高住房建设档次和水平。全市新开工房地产面积650万平方米。

　　（九）加强社会管理，维护社会和谐稳定

　　始终坚持发展是硬道理、稳定是硬任务，发展是第一要务、稳定是第一责任不动摇，全力营造和谐、安全、稳定的社会环境，让广大人民群众安居乐业。

　　抓好基层服务管理。深入推进嘎查村级事务契约化、社区化管理和民主管理工作，提高群众自我服务和发展能力。在全市80%社区建成社区服务中心、卫生服务站和文化服务站，完善服务功能，推动社区服务社会化、产业化。加强非公有制经济组织和新社会组织规范化建设，引导其积极参与经济建设和社会管理。强化企事业单位社会服务和管理责任，全面提升社会服务管理水平。

　　大力化解社会矛盾。加强基层信访工作网络建设，建成各旗县区联合接访中心，建设苏木乡镇、街道办事处综治信访服务中心。进一步拓宽信访渠道，通过网上信访、诉求专线等形式，方便群众反映问题。完善信访工作机制，集中化解各类信访积案和矛盾纠纷，努力把各种不稳定因素化解在基层和萌芽状态。建立信访储备金制度，对特殊困难信访人员实施救助。教育引导群众依法理性表达利益诉求，控制非正常越级上访。

　　切实加强安全生产。强化企业主体责任，狠抓一岗双责和各项措施落实，保障人民群众生命财产安全。完善安全生产联合执法工作机制，对重大安全隐患治理实行逐级挂牌督办。加大矿山、烟花爆竹、危险化学品、交通运输、建筑施工、人员密集场所等重点行业领域隐患排查和专项整治力度，依法关闭不具备安全生产条件的企业，坚决遏制重特大安全生产事故发生。加强食品药品监管，确保食品药品安全。

　　推进平安赤峰建设。完善社会治安防控体系，加强城乡社区警务、群防群治等基层基础建设，增强公共安全和社会治安保障能力。加强社会治安综合治理，依法打击各种违法犯罪活动，维护国家安全和国家利益，保持社会和谐稳定。建立健全对灾害事故和公共安全事件的预警、应急处置体系，增强应急指挥协调能力。加强公共安全宣传教育和应急演练，提高广大群众自我保护意识和应对突发事件能力。全面贯彻落实党的民族宗教政策，依法管理民族宗教事务。支持国防、军队建设，巩固军政军民团结，实现创建全国双拥模范城七连冠。

　　（十）加强政府自身建设，打造廉洁高效政府

　　政府的执行力和落实力是完成十二五规划和今年任务的重要保证，必须恪尽职守，执政为民，强化落实，努力建设人民满意的政府。

　　坚持依法行政。严格按照法定权限和程序行使权利，履行职责。坚持重大事项向人大报告、向政协通报制度，认真办理人大代表意见、建议和政协委员提案。主动听取民主党派、工商联和无党派人士意见，自觉接受人民群众监督和新闻舆论监督。抓好政府法制建设，健全规范性文件监督管理制度，做好行政复议和行政应诉工作。

　　加快转变职能。加强服务型政府建设，建立行为规范、程序合法、运转协调、公正透明的行政管理体制，提高社会管理和公共服务水平。理顺和规范部门职责权限，完善部门之间协调配合机制，解决职能交叉、权责脱节、政出多门问题。减少行政审批事项，简化审批程序，提高行政效率。发展电子政务，推行办公自动化和网上审批。深化政务公开，畅通群众知情、参与、表达和监督渠道。

　　切实转变作风。坚持讲实话、办实事、求实效，杜绝形式主义、弄虚作假和虚报浮夸现象。强化行政效能监察，切实解决行政不作为问题，严肃查处少数干部办事不公、作风粗暴、吃拿卡要、刁难群众等行为，保证令行禁止、政令畅通。严格执行公务接待管理规定，开展公务用车专项治理。精简会议、文件，改进会风、文风。

　　坚持廉洁从政。加强以完善惩治和预防腐败体系为重点的反腐倡廉建设，着力解决人民群众反映强烈的突出问题。针对腐败现象易发多发的领域和环节，进一步加强制度建设和权力制约，从源头上防治腐败。强化对政府投资项目的管理、监察和审计，继续搞好工程建设领域突出问题专项治理。加强对工程招投标、土地出让、产权交易、医药购销、政府采购等领域的监管，严肃查处违法违纪行为，树立风清气正的政府形象。

　　各位代表！我们正处在加快发展的重要时期，把握新形势，抢抓新机遇，推进大发展，是时代赋予我们的重大责任和神圣使命。让我们在市委领导下，团结拼搏，锐意进取，为实现经济社会又好又快发展、开创赤峰更加美好未来而努力奋斗！
